# The heptamer sgRNA targeting the human OCT4 mRNA can upregulate the OCT4 expression

**DOI:** 10.1016/j.bbrep.2021.100918

**Published:** 2021-02-02

**Authors:** Tadasuke Nozaki, Masayuki Takahashi, Tatsuya Ishikawa, Arisa Haino, Mineaki Seki, Hidetomo Kikuchi, Bo Yuan, Masayuki Nashimoto

**Affiliations:** a Department of Clinical Molecular Genetics, Faculty of Pharmacy, Tokyo University of Pharmacy and Life Sciences, Horinouchi 1432-1, Hachioji, Tokyo, 192-0392, Japan; b Research Institute for Healthy Living, Niigata University of Pharmacy and Applied Life Sciences, Higashijima 265-1, Akihaku, Niigata, Niigata, 956-8603, Japan

**Keywords:** Amnion cell, HL60, OCT4, sgRNA, TRUE gene Silencing

## Abstract

TRUE gene silencing is one of the gene suppression technologies. This technology exploits the enzymatic property of the tRNA 3′ processing endoribonuclease tRNase Z^L^, which is that it can cleave a target RNA under the direction of a small guide RNA (sgRNA). We have been working on the development of therapeutic sgRNAs for hematological malignancies. In the course of an experiment to examine the ability of the heptamer-type sgRNA H15792 targeting the OCT4 mRNA to differentiate human amnion stem cells, we observed unexpectedly that the amnion cells exhibited a morphology resembling initialized cells. Here we investigated the effect of H15792 on human HL60 leukemia cells, and found that H15792 can upregulate the OCT4 expression and the expression of alkaline phosphatase in the cells.

## Highlights

• TRUE gene silencing is a gene suppression technology with tRNase Z^L^ and sgRNA.
• We designed and synthesized an sgRNA targeting the OCT4 mRNA.
• The sgRNA induced amnion cells to take a morphology resembling initialized cells.
• The sgRNA upregulated OCT4 and alkaline phosphatase expression in HL60 cells.

## 1 Introduction

tRNase Z^L^-utilizing efficacious gene silencing (TRUE gene silencing) is one of the gene suppression technologies [[1], [2], [3], [4], [5], [6], [7], [8]]. This technology exploits the enzymatic property of the tRNA 3′ processing endoribonuclease tRNase Z^L^, which is that it can cleave a target RNA under the direction of a small guide RNA (sgRNA) by recognizing a pre-tRNA-like or micro-pre-tRNA-like complex formed between the RNA target and the sgRNA [[9], [10], [11], [12], [13], [14], [15]]. Human cells appear to be intrinsically using this property with 5′-half-tRNAs and miRNAs as sgRNAs to modulate gene expression both intra- and inter-cellularly [[16], [17], [18], [19]]. Four types of sgRNA, 5′-half-tRNA, 14-nt linear RNA, hook RNA, and heptamer RNA are known to function as sgRNA.

We have been working on the development of therapeutic sgRNAs for hematological malignancies by focusing on the heptamer-type sgRNA considering cost efficiency. We have shown that heptamer-type sgRNAs designed to target the human BCL2 or WT1 mRNA efficiently induce apoptosis in human leukemia cells and that heptamer-type sgRNAs designed to target the human BCL2 or CCND1 mRNA efficiently trigger apoptosis in human myeloma cells [[20], [21], [22]]. Twenty sgRNAs with efficient capability to induce apoptosis in leukemia and/or myeloma cells have also been found from heptamer-type sgRNA library screening [[23], [24], [25]]. Furthermore, we have found that a heptamer-type sgRNA can shift macrophages toward the M1 state and efficiently suppress human myeloma cell growth in immunocompromised mice [26].

Allogeneic hematopoietic stem cell transplantation is one of the therapies to treat hematological malignancies, but a high incidence of graft-versus-host disease (GVHD) remains a major obstacle [27]. Although patients with steroid-resistant GVHD, in particular, show a poor prognosis, its treatment with mesenchymal stem cells (MSCs) is expected to remedy this situation [28]. MSCs can not only be obtained from various tissues such as bone marrow and umbilical cord, but also be derived from induced pluripotent stem cells (iPSCs) [29]. The iPSC-derived MSCs may become a stable and homogeneous off-the-shelf source for the GVHD cell therapy. Although iPSCs were originally generated by expressing four genes, OCT4, SOX2, KIF4, and MYC in fibroblasts, it has also been demonstrated that activation of the endogenous OCT4 gene can solely induce pluripotency and that expression of the exogenous miR-302/367 cluster can reprogram somatic cells to pluripotency [[30], [31], [32]].

In the course of an experiment to examine the ability of the heptamer-type sgRNA H15792 targeting the OCT4 mRNA to differentiate human amnion stem cells, we observed unexpectedly that the amnion cells exhibited a morphology resembling initialized cells. In this paper, we investigated the effect of H15792 on human HL60 leukemia cells, and found that H15792 can upregulate the OCT4 expression and the expression of alkaline phosphatase in the cells.

## 2 Materials and methods

### 2.1 Synthetic RNA

Fully 2′-*O*-methylated, 5′- and 3′-phosphorylated sgRNAs, H15792 (5′-pCCUGGCCp-3′), H16048 (5′-pCCGGGCCp-3′), and H14038 (5′-pCUGCUUUp-3′), were synthesized with a DNA/RNA synthesizer and purified by high-performance liquid chromatography by Nippon Bioservice (Asaka, Saitama, Japan). Although we usually use naked heptamer-type sgRNAs at a final concentration of 1 μM [[20], [21], [22],[24], [25], [26]], they can also work at a lower final concentration of 0.2 μM [8].

### 2.2 Preparation of primary cultured amnion cells

Fetal membranes were prepared aseptically from placenta during normal parturition by Cesarean section as described previously [33,34]. Briefly, after carefully removing of decidua with forceps, the amniochorion tissues were washed with ice-cold PBS+ (phosphate buffered saline containing 150 units/ml penicillin G sodium, 150 μg/ml streptomycin sulfate, 0.375 μg/ml amphotericin B, 150 μg/ml kanamycin and 20 μg/ml gentamicin sulfate (GibcoBRL, MD, USA)) several times to remove blood and clots.

The amnion tissues were separated from the fresh amniochorion tissues and used to prepare amnion cells according to the methods described previously [33,34]. Briefly, amnion tissue was washed again with PBS+ and cut into pieces (~1 cm^2^). The pieces up to 5 g were incubated in 50 ml of calcium- and magnesium-free Hank's balanced salt solution (Invitrogen, CA, USA) containing 0.25% trypsin (GibcoBRL) and 0.04% collagenase type I (Invitrogen) for 45 min at 37 °C. This digest was strained and the eluate was collected. These procedures were repeated one more time. The eluates were pelleted by centrifugation at 700×*g* for 5 min at 4 °C to obtain amnion cells. Written informed consent was obtained from the patients at the time of surgery, and this study was approved by the Institutional Review Board committee of Tokyo University of Pharmacy and Life Science. Since fetal membranes are regarded as medical waste, their use is generally accepted.

### 2.3 Microscopy

The amnion cells were re-suspended in culture medium composed of 80% 1:1 mixture of Dulbecco's modified Eagle medium and Ham's F-12 medium (GibcoBRL) supplemented with 0.244% NaHCO_3_, 20% heat-inactivated fetal bovine serum (Bio-Whittaker Inc. MD, USA) and antibiotics (150 units/ml penicillin G sodium, 150 μg/ml streptomycin sulfate, 0.375 μg/ml amphotericin B, 150 μg/ml kanamycin and 20 μg/ml gentamicin sulfate), and cultured in a humidified atmosphere with 5% CO_2_ at 37 °C.

The amnion cells were seeded at 5 × 10^5^ cells/500 μl/well into 24-well plates coated with type I collagen (Iwaki, Tokyo, Japan), and then cultured without serum in the absence or presence of 1 μM of naked heptamer-type sgRNA H15792 or H16048 for 4 days. Morphological change of the cells was examined with a phase-contrast microscope, Axiovert 200 M (Carl Zeiss, Jena, Germany).

### 2.4 Real time-PCR

The human leukemia cell line HL60 was cultured in RPMI-1640 media (Wako, Osaka, Japan) supplemented with 10% fetal bovine serum (MP Biomedicals Japan, Tokyo, Japan) and 1% penicillin-streptomycin (Invitrogen Japan, Tokyo, Japan) at 37 °C in 5% CO_2_ humidified incubator. The cells were seeded at 10^4^ cells/500 μl/well on a 24-well plate, and cultured in the absence or presence of 1 μM of naked heptamer-type sgRNA H15792 or H16048 for 1–5 days.

Total RNA was extracted from the HL60 cells with RNAiso Plus (Takara Bio, Shiga, Japan). The levels of OCT4 and GAPDH mRNAs were measured by real-time PCR using a ReverTra Ace qPCR RT Master Mix (Toyobo, Osaka, Japan) and a SYBR Premix Ex *Taq*II (Takara Bio) with a Thermal Cycler Dice Real Time System (Takara Bio) under the standard conditions. The OCT4 mRNA level was normalized against the ACTB or GAPDH mRNA level. The primer pair for the OCT4 mRNA was 5′-CGTGAAGCTGGAGAAGGAGAAGCT-3′ and 5′- CAAGGGCCGCAGCTTACACATGTTC-3′, and that for the GAPDH mRNA was 5′-CCCACTCCTCCACCTTTGAC-3′ and 5′-ACCCTGTTGCTGTAGCCAAA-3′. The primer pair for the ACTB mRNA was 5′-CTGGAACGGTGAAGGTGACA-3′ and 5′-AAGGGACTTCCTGTAACAACGCA-3′, or 5′-ACAATGTGGCCGAGGACTTT-3′ and 5′-TGTGTGGACTTGGGAGAGGA-3′.

### 2.5 Alkaline phosphatase staining

HL60 cells were seeded at 10^4^ cells/500 μl/well on a 24-well plate, and cultured as above in the absence or presence of 0.5 μM of naked heptamer-type sgRNA H15792 or H14038 for 4 or 5 days. Then, the cells were stained with Alkaline Phosphatase Live Stain (Thermo Fisher Scientific K.K.), and the data of fluorescence from the existence of alkaline phosphatase in the cell was obtained with the confocal laser scanning microscope FV1000-D (Olympus Corporation, Tokyo, Japan). The mean fluorescence intensity per cell was analyzed with an image processing program, ImageJ v1.53e (the National Institutes of Health).

## 3 Results

### 3.1 The sgRNA targeting the OCT4 mRNA can make amnion cells take a morphology resembling initialized cells

**Fig. 1:**
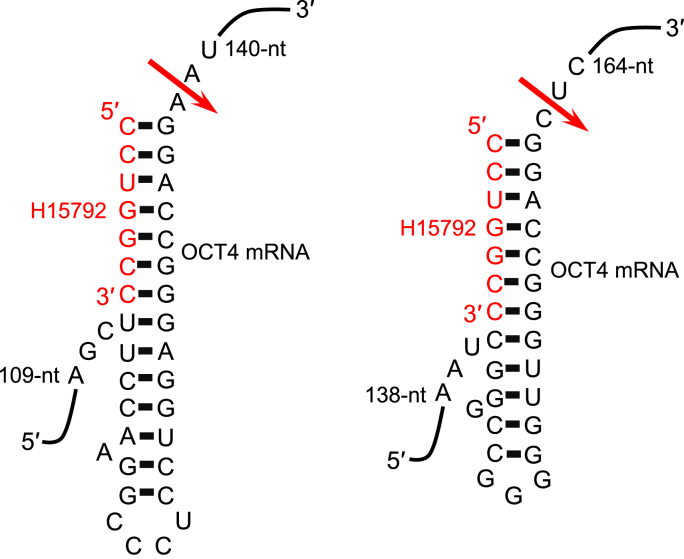
The heptamer-type sgRNA H15792 targeting the human OCT4 mRNA. Possible secondary structures of the complexes between H15792 and the OCT4 mRNA formed at two different sites. An arrow denotes the expected cleavage site by tRNase Z^L^. The nucleotide numbering system starts with one for the A of the initiation codon.

**Fig. 2:**
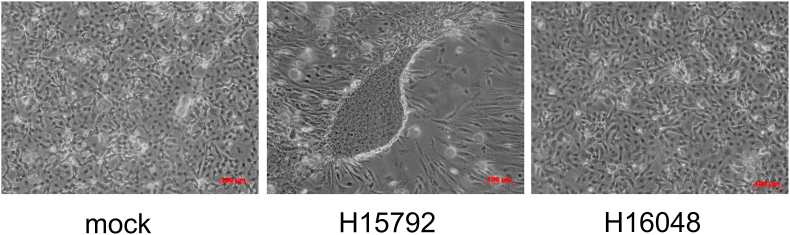
Microscopic images of human amnion cells. The cells were cultured in the absence or presence of heptamer-type sgRNA H15792 or H16048 for 4 days, and analyzed with a phase-contrast microscope.

In order to examine if sgRNA has an ability to differentiate human amnion stem cells by downregulating the OCT4 expression, we designed and synthesized a heptamer-type sgRNA, H15792, targeting the human OCT4 mRNA at its two sites (Fig. 1). Amnion cells were cultured without serum in the presence of the naked sgRNA H15792. And, by the 4th day, we observed unexpectedly that the cells formed 3 colonies, all of which exhibited a morphology resembling initialized cells (Fig. 2) [30]. A control heptamer-type sgRNA, H16048, did not show this ability. Although we examined amnion cells from 6 persons, this transformation was not observed in the other 5 cases.

### 3.2 The sgRNA H15792 can upregulate the OCT4 expression in HL60 cells

**Fig. 3:**
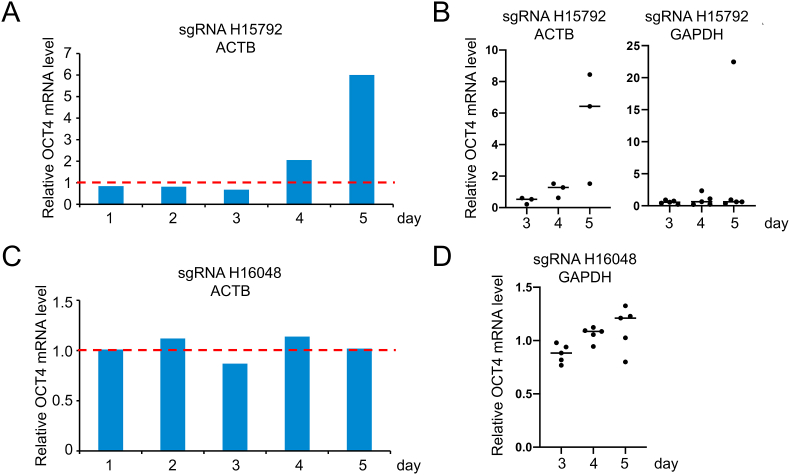
Effects of the heptamer-type sgRNAs H15792 (A, B) and H16048 (C, D) on the OCT4 mRNA level in HL60 cells. The OCT4 mRNA levels in the cells treated with sgRNA for 1–5 days were quantitated by real-time PCR, and their values relative to those in the untreated cells are shown. The OCT4 mRNA levels were normalized against the ACTB or GAPDH mRNA levels. (B, D) The number of dots represents that of independent experiments, and bars denote median values.

Although the OCT4 mRNA level in the amnion cells should have been measured to elucidate the mechanism of their transformation, we instead quantitated it in the human HL60 leukemia cells since the primary cultured amnion cells were very limited resources. The OCT4 mRNA levels in HL60 cells were measured 1–5 days after the addition of the naked heptamer-type sgRNA H15792. Its levels continued to decrease to up to 68% until day 3, but intriguingly were boosted by 6-fold by day 5 (Fig. 3A).

Two more series of the experiments (one with three sets and the other with five sets) were performed under the same conditions by focusing on the change in the OCT4 mRNA levels at 4–5 days after the sgRNA addition. The OCT4 expression was upregulated by > 2-fold at six data points, and boosted by > 20-fold at day 5 in one set (Fig. 3B). The OCT4 mRNA levels were not upregulated significantly by adding the control sgRNA H16048 at any data points (Fig. 3C and D).

### 3.3 The sgRNA H15792 can upregulate the expression of alkaline phosphatase in HL60 cells

**Fig. 4:**
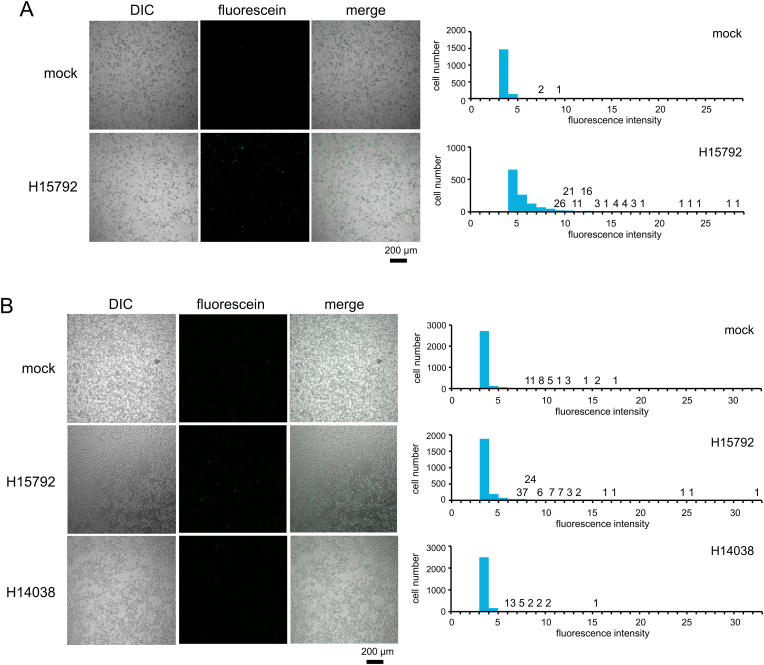
Effects of the heptamer-type sgRNAs H15792 on the expression of alkaline phosphatase in HL60 cells. The cells were cultured in the absence or presence of heptamer-type sgRNA H15792 for 4 days (A), or in the absence or presence of heptamer-type sgRNA H15792 or H14038 for 5 days (B). The data of fluorescence from the existence of alkaline phosphatase in the cell was obtained with a confocal laser scanning microscope and analyzed with ImageJ. The number of cells that emitted the fluorescence of the same intensity range was presented as a histogram. The total cell numbers counted in “mock” and “H15792” (A) were 1611 and 1258, respectively, and those in “mock”, “H15792”, and “H14038” (B) were 2991, 2291, and 2714, respectively. A number above an intensity range denotes a cell count. DIC, differential interference contrast microscopic image; fluorescein, fluorescence image.

Next, we examined HL60 cells for the expression of alkaline phosphatase, which is known to be a marker of pluripotent stem cells [30], in the presence of the sgRNA H15792. Obvious upregulation of alkaline phosphatase was observed in HL60 cells treated with H15792 for 4 days (Fig. 4A). While fluorescence from most of the untreated HL60 cells was in its intensity range 3–4, its distribution in the H15792-treated cells had a peak at the intensity range 4–5 and about a half of the cells emitted stronger fluorescence. Of note, the intensity of fluorescence from five cells was higher than 22. However, we were not able to detect any colonies that take a morphology resembling initialized cells.

The upregulation of alkaline phosphatase was not observed in the cells treated with a control sgRNA, H14038, for 5 days (Fig. 4B). The fluorescence from the majority (92%) of the cells was in its intensity range 3–4, and its distribution was similar to that in the untreated cells. On the other hand, the fluorescence from 18% of the H15792-treated cells were in its intensity ranges >4, and its intensity from three cells was higher than 24.

## 4 Discussion

The morphological change of the amnion cells by H15792 occurred in one of the six independent experiments, suggesting that certain genetic and/or environmental factors of the cells may affect their transcriptomic change that leads to a successful morphological transformation. Even in the case that the transformation occurred, only three colonies were formed, suggesting that only three cells among 5 × 10^5^ amnion cells were initialized by the sgRNA. The frequency of the colony formation was ~30-fold less than that in the experiments with Yamanaka factors, in which ~10 embryonic stem cell-like colonies were formed from 5 × 10^4^ human fibroblasts [30].

The measurement of the OCT4 mRNA levels in HL60 cells showed that its upregulation (>2-fold) in the presence of H15792 occurred two and four times at day 4 and day 5, respectively, in nine sets of the independent experiments (Fig. 3A and B). Although we did not quantitate each OCT4 mRNA level in a single cell, its distribution pattern should be similar to that of the alkaline phosphatase expression (Fig. 4) since the OCT4 upregulation triggers the expression of alkaline phosphatase [35]. From the observations in HL60 cells, we also inferred that the morphological change of the amnion cells was induced through upregulation of the OCT4 expression by the heptamer-type sgRNA H15792.

At present, we do not know how H15792 targeting the OCT4 mRNA can boost the OCT4 expression after downregulating it for 3 days. The slight decrease in the OCT4 mRNA level might somehow induce chromatin remodeling in the OCT4 promoter/enhancer region [31]. Alternatively, H15792 might happen to suppress genes that suppress OCT4 like miR-302/367 [32].

Our current observation suggests that the heptamer-type sgRNA H15792 alone might be able to generate iPSCs from fibroblasts. And these iPSCs would be able to be used to generate various clinically useful cells including MSCs for the GVHD treatment. Furthermore, if upregulation of gene expression by heptamer-type sgRNA can occur in a wide spectrum of genes, we might be able to use the heptamer-type sgRNA to treat diseases linked to low expression of some specific genes.

## Funding

This work was supported by Adaptable and Seamless Technology Transfer Program through Target-driven R&D, Japan Science and Technology Agency.

## CRediT authorship contribution statement

**Tadasuke Nozaki:** Conceptualization, Investigation, Writing - review & editing. **Masayuki Takahashi:** Investigation, Methodology, Writing - review & editing. **Tatsuya Ishikawa:** Investigation, Formal analysis, Writing - review & editing. **Arisa Haino:** Investigation, Writing - review & editing. **Mineaki Seki:** Investigation, Writing - review & editing. **Hidetomo Kikuchi:** Resources, Supervision, Writing - review & editing. **Bo Yuan:** Resources, Supervision, Writing - review & editing. **Masayuki Nashimoto:** Conceptualization, Methodology, Resources, Supervision, Project administration, Funding acquisition, Writing - original draft, Writing - review & editing.

## Declaration of competing interest

The authors declare the following financial interests/personal relationships which may be considered as potential competing interests. The author MN is an advisor of Veritas In Silico Inc., and owns stock of the company.
